# Exploring the safety, effect on the tumor microenvironment, and efficacy of itacitinib in combination with epacadostat or parsaclisib in advanced solid tumors: a phase I study

**DOI:** 10.1136/jitc-2021-004223

**Published:** 2022-03-14

**Authors:** Aung Naing, John D Powderly, John J Nemunaitis, Jason J Luke, Aaron S Mansfield, Wells A Messersmith, Solmaz Sahebjam, Patricia M LoRusso, Ignacio Garrido-Laguna, Lance Leopold, Ryan Geschwindt, Kai Ding, Michael Smith, Jordan D Berlin

**Affiliations:** 1 Department of Investigational Cancer Therapeutics, MD Anderson Cancer Center, Houston, Texas, USA; 2 Cancer Research Clinic, Carolina Biooncology Institute, Huntersville, North Carolina, USA; 3 University of Toledo College of Medicine, Toledo, Ohio, USA; 4 Division of Hematology/Oncology, University of Pittsburgh Medical Center, Pittsburgh, Pennsylvania, USA; 5 Oncology, Mayo Clinic, Rochester, Minnesota, USA; 6 School of Medicine, University of Colorado, Aurora, Colorado, USA; 7 Clinical Research Unit, Moffitt Cancer Center, Tampa, Florida, USA; 8 Yale School of Medicine, Yale Cancer Center, New Haven, Connecticut, USA; 9 University of Utah School of Medicine, Huntsman Cancer Institute, Salt Lake City, Utah, USA; 10 Immuno-Oncology, Incyte Corporation, Wilmington, Delaware, USA; 11 Biostatistics, Incyte Corporation, Wilmington, Delaware, USA; 12 Division of Hematology/Oncology, Vanderbilt University, Nashville, Tennessee, USA

**Keywords:** tumor microenvironment, biomarkers, tumor, clinical trials as topic, drug therapy, combination, immunomodulation

## Abstract

**Background:**

This phase I multicenter study was designed to evaluate the safety, tolerability, efficacy, and translational effects on the tumor microenvironment of itacitinib (Janus-associated kinase 1 (JAK1) inhibitor) in combination with epacadostat (indoleamine 2,3-dioxygenase 1 (IDO1) inhibitor) or parsaclisib (phosphatidylinositol 3-kinase δ (PI3Kδ) inhibitor).

**Methods:**

Patients with advanced or metastatic solid tumors were enrolled and received itacitinib (100–400 mg once a day) plus epacadostat (50–300 mg two times per day; group A), or itacitinib (100–400 mg once a day) plus parsaclisib or parsaclisib monotherapy (0.3–10 mg once a day; group B).

**Results:**

A total of 142 patients were enrolled in the study. The maximum tolerated dose was not reached for either the combination of itacitinib plus epacadostat (n=47) or itacitinib plus parsaclisib (n=90). One dose-limiting toxicity of serious, grade 3 aseptic meningitis was reported in a patient receiving itacitinib 300 mg once a day plus parsaclisib 10 mg once a day, which resolved when the study drugs were withdrawn. The most common treatment-related adverse events among patients treated with itacitinib plus epacadostat included fatigue, nausea, pyrexia, and vomiting, and for patients treated with itacitinib plus parsaclisib were fatigue, pyrexia, and diarrhea. In the itacitinib plus epacadostat group, no patient had an objective response. Among patients receiving itacitinib 100 mg once a day plus parsaclisib 0.3 mg once a day, three achieved partial response for an objective response rate (95% CI) of 7.1% (1.50 to 19.48). Treatment with itacitinib plus epacadostat demonstrated some increase in tumor CD8^+^ T cell infiltration and minor changes in six plasma proteins, whereas treatment with itacitinib plus high-dose parsaclisib resulted in downregulation of 20 plasma proteins mostly involved in immune cell function, with no observed change in intratumoral CD8^+^ T cell infiltration.

**Conclusion:**

Adverse events with JAK1 inhibition combined with either IDO1 or PI3Kδ inhibition were manageable, but the combinations demonstrated limited clinical activity or enhancement of immune activation in the tumor microenvironment.

**Trial registration number:**

NCT02559492.

## Introduction

Immune inhibitory pathway blockade is an important therapeutic strategy for cancer treatment, with promising clinical responses observed using antibodies blocking cytotoxic T lymphocyte antigen (CTLA)-4, programmed death-1 or programmed death-ligand 1 (PD-(L)1).^1 2^ PD-1 or CTLA-4 inhibition (alone or in combination) is more effective in tumors that are immunogenic, with T cell infiltration or higher mutation burden.^2^ Although these agents have antitumor activity, multiple mechanisms are present within the tumor microenvironment (TME), suggesting that combination therapies may improve therapeutic effects.^1 3^

Janus-associated kinase (JAK)/signal transducer and activator of transcription (STAT) signaling is known to reduce antitumor responses by increasing the number of immunosuppressive and tumor-promoting myeloid-derived suppressor cells (MDSCs) and tumor-associated macrophages (TAMs).^4^ In the murine pancreatic PAN02 ductal adenocarcinoma preclinical model, the JAK1 inhibitor itacitinib (INCB039110) reduced tumor growth and increased the overall number and activity of CD4^+^ and CD8^+^ effector cells, along with a concomitant decrease in suppressor cells, including regulatory T cells (Tregs), TAMs, and MDSCs.^5^ Within the TME, JAK inhibition led to a decrease in proinflammatory cytokines and chemokines key for recruitment and activity of Tregs.^5^

Indoleamine 2,3-dioxygenase 1 (IDO1) contributes to immune suppression by catalyzing tryptophan breakdown to kynurenine, which results in blockade of T cell activation and effector function, induces T cell apoptosis, and converts naïve T cells to forkhead box protein 3 (FoxP3)^+^ Tregs.^6^ Epacadostat (INCB024360) is a potent and selective small-molecule inhibitor of IDO1 originally assessed in the PAN02 model, where it was shown to induce T cell-dependent antitumor immunity; this activity was related to the capacity to promote dendritic cell function and maturation and decreased numbers of Tregs.^7^ In a phase I first-in-human study, epacadostat was generally well tolerated at dose levels (≥100 mg two times per day) predicted to yield clinically meaningful suppression of IDO1 activity in patients with advanced solid tumors.^8^

Phosphatidylinositol 3-kinase δ (PI3Kδ) plays a role in promoting growth of B cell malignancies,^9 10^ but has also been reported to be involved in solid tumors. For instance, elevated expression of PI3Kδ in liver cancer may contribute to tumor progression.^11^ Inhibition of PI3Kδ leads to decrease in Tregs and MDSCs, a concomitant increase in effector T cell activity, and reduced growth of multiple tumor types, including 4T1 breast cancer, Lewis lung carcinoma, B16 melanoma, and EL4 thymoma.^12^ Disrupting Treg and MDSC function via PI3Kδ inhibitors thus has the potential to enhance antitumor immunity in solid tumors.^12 13^ Single-agent PI3Kδ inhibitor parsaclisib (INCB050465) treatment in the PAN02 cancer model increased the number of infiltrating CD8^+^ cells and reduced tumor volume.^5^

Immunotherapy combination studies in the PAN02 model demonstrated enhanced tumor growth control when JAK inhibition was combined with either epacadostat, parsaclisib, or anti-PD-L1 antibody. In addition, specific combinations increased the number of intratumoral effector cells or activation phenotype of immune cells.^5^ In preclinical melanoma models, IDO1 inhibition synergizes with either anti-CTLA-4 or anti-PD-(L)1 in delaying tumor growth and increasing survival.^14 15^ Based on preclinical findings, JAK1 combined with IDO1 or PI3Kδ inhibition may result in greater immunomodulatory effects than either agent alone.

Here, we report the results from a phase I study evaluating the safety, tolerability, effect on the TME, and efficacy of itacitinib in combination with epacadostat or parsaclisib in patients with advanced or metastatic solid tumors. The translational hypothesis tested was that blockade of excessive JAK/STAT signaling would selectively enhance the antitumor immune response by decreasing intratumoral MDSCs and Tregs, while increasing the number of activated CD8^+^ effector T cells. Pharmacodynamic effects on the TME and potential association with response to study treatment were evaluated by paired biopsy and plasma proteomic analysis.

## Methods

### Study design

**Figure 1:**
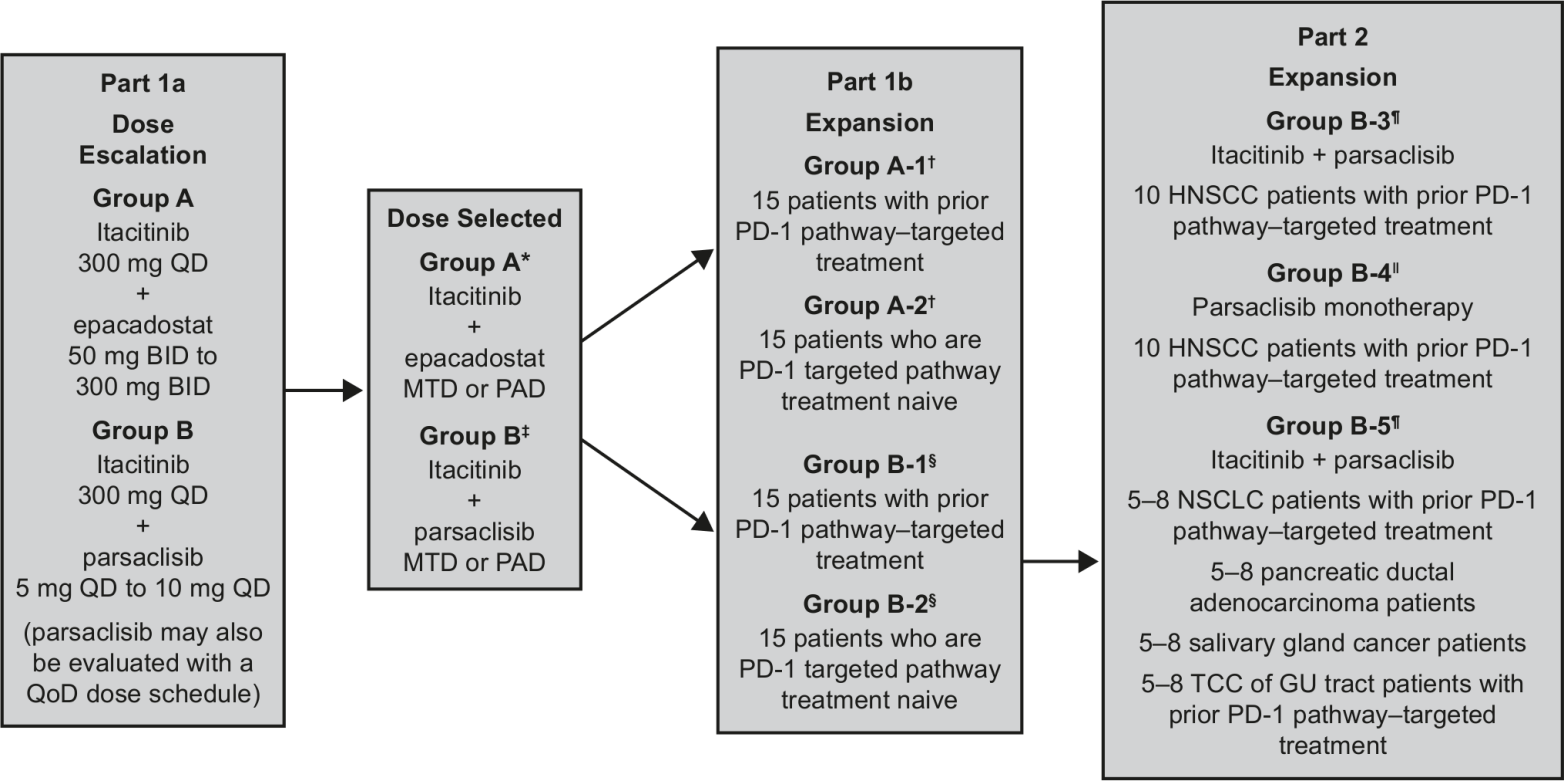
Study design. *Group A included three dose levels: itacitinib 300 mg once a day plus epacadostat 50 mg two times per day, itacitinib 300 mg once a day plus epacadostat 100 mg two times per day, and itacitinib 300 mg once a day plus epacadostat 300 mg two times per day. †Treatment for groups A-1 and A-2 was itacitinib 300 mg once a day plus epacadostat 300 mg two times per day. ‡Group B included seven dose levels: itacitinib 300 mg once a day plus parsaclisib 2.5 mg once every other day, itacitinib 300 mg once a day plus parsaclisib 5 mg once a day, itacitinib 300 mg once a day plus parsaclisib 10 mg once a day, itacitinib 100 mg once a day plus parsaclisib 0.3 mg once a day, itacitinib 100 mg once a day plus parsaclisib 1 mg once a day, itacitinib 300 mg once a day plus parsaclisib 0.3 mg once a day, and itacitinib 300 mg once a day plus parsaclisib 1 mg once a day. §Treatment for groups B-1 and B-2 was itacitinib 300 mg once a day plus parsaclisib 10 mg once a day. ¶Treatment for groups B-3 and B-5 was itacitinib 100 mg once a day plus parsaclisib 0.3 mg once a day. ǁTreatment for group B-4 was parsaclisib 0.3 mg once a day monotherapy; one patient in group B-4 had itacitinib 100 mg once a day added, per protocol, due to disease progression. All patients receiving parsaclisib plus itacitinib (except parsaclisib 0.3 mg once a day plus itacitinib 100 mg once a day) were required to receive a standard *Pneumocystis jirovecii* prophylaxis regimen determined by the investigator. BID, two times per day; GU, genitourinary; HNSCC, head and neck squamous cell carcinoma; MTD, maximum tolerated dose; NSCLC, non-small cell lung cancer; PAD, pharmacologically active dose; PD-1, programmed cell death-1; QD, once a day; QoD, once every other day; TCC, transitional cell carcinoma.

This multicenter, open-label, non-randomized, parallel-assessed, phase I platform study was conducted at 10 centers in the USA. The study was designed in three parts (part 1a: itacitinib plus epacadostat or parsaclisib dose escalation; part 1b: itacitinib plus epacadostat or parsaclisib dose expansion; and part 2: itacitinib plus parsaclisib or parsaclisib monotherapy) (figure 1). Patients provided informed consent before enrollment.

Dose escalation (part 1a) was conducted using a 3+3 design to determine the maximum tolerated dose (MTD) or pharmacologically active dose, and dose recommendation for part 1b dose expansion for each combination. MTD was defined as the highest dose at which fewer than one-third of patients experienced a dose-limiting toxicity (DLT). Two parallel treatment arms explored itacitinib in combination with either epacadostat (group A) or parsaclisib (group B) administered over 21-day cycles. Patients in group A were treated with itacitinib (300 or 400 mg) once a day plus epacadostat (50 mg, 100 mg, or 300 mg) two times per day. Patients in group B were treated with itacitinib (100 mg, 300 mg, or 400 mg) once a day plus parsaclisib (0.3 mg, 1, 2.5 mg, 5 mg, or 10 mg) once a day; in a protocol amendment during the course of the study, patients who received parsaclisib doses ≥5 mg once a day were switched from once a day to once weekly dosing at cycle 4, day 1. Part 1b was a safety expansion at the doses selected in part 1a and enrolled patients based on treatment history with a PD-(L)1-targeted agent (groups A-1 and B-1) or who were PD-(L)1 treatment-naïve (groups A-2 and B-2).

During the course of the study, emerging reports suggested a differential effect of PI3Kδ inhibition between effector T cells and Tregs, with lower doses of PI3Kδ inhibition being more active on Treg inhibition. In a protocol amendment, part 2 was added to include additional expansion cohorts that evaluated lower doses of itacitinib and parsaclisib. Patients with progressive disease (PD) on standard therapies were enrolled into three expansion groups to evaluate treatment with itacitinib 100 mg once a day and parsaclisib 0.3 mg once a day. Two of the expansion cohorts compared parsaclisib monotherapy (group B-4) with itacitinib plus parsaclisib (group B-3) in patients with head and neck squamous cell carcinoma (HNSCC) who had received prior PD-L1-targeted therapy. One expansion cohort evaluated itacitinib plus parsaclisib (group B-5) in patients with non-small cell lung cancer (NSCLC) or genitourinary (GU) tract transitional cell carcinoma (TCC) who had prior PD-(L)1-targeted therapy and in patients with pancreatic ductal adenocarcinoma (PDAC) or salivary gland cancer. Patients who received parsaclisib monotherapy (group B-4) were allowed to add itacitinib on PD. Study treatment could continue while patients derived benefit and had not met withdrawal criteria.

### Patients

The study enrolled men and women ≥18 years of age, who had an Eastern Cooperative Oncology Group performance status of ≤1, and were willing to provide a baseline and on-treatment tumor biopsy specimen. Patients were enrolled into the subsequent treatment arms based on inclusion criteria described in the following.

Part 1a enrolled patients with histologically or cytologically confirmed advanced or metastatic solid tumors that progressed following prior standard therapy. Part 1b enrolled patients with endometrial cancer, gastric cancer, HNSCC, melanoma, microsatellite unstable colorectal cancer, NSCLC, PDAC, renal cell carcinoma, triple-negative breast cancer, or GU tract TCC. Part 2 enrolled patients with HNSCC, NSCLC, PDAC, salivary gland cancer, or GU tract TCC who had disease progression after available therapies for advanced or metastatic disease that are known to confer clinical benefit, or were intolerant to or refused standard treatment.

### Study endpoints and assessments

The primary endpoint was safety and tolerability as assessed by monitoring the frequency, duration, and severity of adverse events (AEs). Treatment-emergent adverse events (TEAEs) were defined as any AE reported either for the first time or worsening of a pre-existing event after the first dose of the study drug and until 30 days after the last dose of the study drug. TEAEs were summarized using Medical Dictionary for Regulatory Activities (version 19.1) preferred terms, and severity was graded by the National Cancer Institute Common Terminology Criteria for Adverse Events version 4.03 criteria.

Secondary study endpoints included objective response rate (ORR) determined by radiographic assessments per Response Evaluation Criteria in Solid Tumors (RECIST) version 1.1, progression-free survival (PFS) and duration of response (DOR) determined by the investigator per RECIST version 1.1, and percentage of responders. Overall response was evaluated at each postbaseline radiological assessment. An increase in the number of tumor-infiltrating lymphocytes (TIL) or the ratio of CD8^+^ lymphocytes to Tregs infiltrating tumor post-treatment (itacitinib plus epacadostat or parsaclisib) versus baseline was used to calculate the percentage of TIL responders.

For assessment of treatment effects on TILs, biopsy samples were collected at baseline (screening or predose cycle 1) and on treatment (between weeks 3 and 5). Biopsies performed early in the study had a high failure rate for evaluable tissue for analysis by immunohistochemistry (IHC). To improve the rate of successful biopsies submitted to the central laboratory vendor for IHC analysis, a protocol amendment required fresh biopsies to be performed at baseline, formalin fixation and paraffin embedding of tumor tissue to be performed at the study site, and H&E stain to be performed and assessed by a local pathologist first to determine if the sample was acceptable for analysis. Acceptable biopsies were required to contain at least 20% tumor content and be free of embedding artifacts; the option was given to rebiopsy if insufficient tissue was obtained.

### Correlative translational studies

In part 1 of the study, IHC was performed using two seven-color, six-plex Multiplex assays (PerkinElmer, Waltham, Massachusetts). Paired baseline and on-treatment biopsy samples were analyzed for CD3, CD8, CD20, CD45RO, FoxP3, cytokeratin (CK), and nuclear counterstain (4′,6-diamidino-2-phenylindole (DAPI)) in panel 1, and CD68, IDO1, PD-L1, pSTAT3, Ki67, CK, and DAPI in panel 2. Cell types of interest in tumor versus stromal (non-tumor) regions and a composite of the total tissue section were quantified (cells/mm^2^) separately using the inForm Advanced Image Analysis (PerkinElmer). For part 2, IHC was performed using a four-plex Multiplex assay (Indivumed, Hamburg, Germany). Paired baseline and on-treatment biopsy samples were analyzed for CD3, CD8, FoxP3, and panCK. Cell densities were quantified separately in tumor and stromal regions and as a composite of the total tissue section by OracleBio (Lanarkshire, Scotland, UK) using the HALO AI digital pathology software.

Modulation of plasma proteins was determined using Multiplex Proximity Extension Assay (Olink Proteomics, Watertown, Massachusetts). Using a matched pair of antibodies coupled to unique, partially complementary oligonucleotides, protein biomarkers were identified and quantified using real-time PCR. Paired baseline and on-treatment samples were evaluated. Plasma kynurenine levels were analyzed by liquid chromatography with tandem mass spectrometry (Worldwide Clinical Trials, Research Triangle Park, North Carolina).

### Statistical analysis

All enrolled patients who received ≥1 dose of the study drug were included in the safety and efficacy evaluable population. Descriptive statistics were used to summarize TEAEs, DLTs, vital signs, ECGs, and clinical laboratory blood and urine data. The exact method for binomial distributions was used to calculate the 95% CI for the proportion of responders (patients with an overall response of complete response (CR) or partial response (PR) at any postbaseline visit). The Brookmeyer and Crowley’s method was used to calculate the Kaplan-Meier estimate of the median PFS. The Kaplan-Meier method was used to calculate the DOR of patients who achieved a response, with median DOR and 95% CI estimated.

For translational correlative studies, within each treatment group, the Wilcoxon matched-pairs signed-rank test (GraphPad Prism version 7.02, GraphPad Software, San Diego, California) was used to compare CD8^+^ and FoxP3^+^ cell infiltration into the tumor region between baseline and on-treatment samples. A patient demonstrating ≥50% increase in CD8^+^ to FoxP3^+^ cell ratio in tumor compartment was classified a TIL responder, with changes deemed significant at p<0.05. For plasma protein analysis, baseline versus on-treatment samples were compared using a paired t-test, with changes considered significant at a false discovery rate p value of <0.05 and a log_2_ fold change >0.4 or <−0.4.

Up to 159 patients were anticipated to be enrolled in the study. In part 1a, with a planned total enrollment of ~24–54 patients, 3–6 patients were enrolled in each dose level depending on the occurrence of DLTs. In part 1b, with a planned enrollment of 30 patients in each treatment group (groups A-1/A-2 and B-1/B-2; total of 60 patients), there was ≥90% chance of observing a toxicity (with a true event rate of >7.4%) and 83.6% probability of observing ≥11 TIL responders within each expansion cohort of 15 patients (assuming the true TIL response rate was 80%). In part 2, with three separate expansion cohorts in select tumor types (planned enrollment total of ~45 patients), there was 85% probability of observing ≥6 of 10 patients with a biomarker in groups B-3 and B-4, and 81% probability of observing ≥16 of 25 patients with a biomarker in group B-5 (assuming the true patient biomarker positive rate was 70%).

## Results

### Patient characteristics and disposition

A total of 142 patients were enrolled in the study: 47 patients in all of group A (itacitinib plus epacadostat; 12 in group A and 35 in groups A-1 and A-2) and 95 patients in all of group B (itacitinib plus parsaclisib; 42 in group B, 23 in groups B-1 and B-2 (high-dose parsaclisib), and 30 in groups B-3, B-4, and B-5 (low-dose parsaclisib)). Patient demographics and baseline characteristics are summarized in table 1. Nearly all patients had received systemic therapy before enrolling into the study. The most common tumor types included pancreatic (48.6% in groups A-1 and A-2), endometrial adenocarcinoma (30.4% in groups B-1 and B-2), and HNSCC (23.3% in groups B-3, B-4, and B-5).

**Table 1:** Summary of demographic and baseline characteristics: group A and B (all enrolled patients)

Variable	Group A: itacitinib+epacadostat		Group B: itacitinib+parsaclisib		
	Part 1a Dose-escalation Group A (n=12)	Part 1b Dose-expansion Groups A-1 and A-2 (n=35)	Part 1a Dose-escalation Group B (n=42)	Part 1b Dose-expansion Groups B-1 and B-2 (n=23)	Part 2 Dose-expansion Groups B-3, B-4, and B-5 (n=30)
Median age (range), years	63.5 (40–74)	62.0 (43–85)	61.0 (37–77)	60.0 (35–85)	62.5 (28–78)
Male, n (%)	5 (41.7)	16 (45.7)	17 (40.5)	8 (34.8)	19 (63.3)
Race, n (%)					
White/Caucasian	10 (83.3)	29 (82.9)	34 (81.0)	18 (78.3)	24 (80.0)
Black/African-American	2 (16.7)	3 (8.6)	6 (14.3)	2 (8.7)	2 (6.7)
Other	0 (0)	3 (8.6)	2 (4.8)	3 (13.0)	4 (13.3)
ECOG PS, n (%)					
0	4 (33.3)	10 (28.6)	8 (19.0)	4 (17.4)	7 (23.3)
1	8 (66.7)	25 (71.4)	34 (81.0)	19 (82.6)	23 (76.7)
Prior therapy, n (%)					
Radiotherapy	6 (50.0)	19 (54.3)	26 (61.9)	14 (60.9)	24 (80.0)
Surgery	10 (83.3)	29 (82.9)	37 (88.1)	19 (82.6)	26 (86.7)
Systemic therapy	12 (100)	35 (100)	41 (97.6)	23 (100)	29 (96.7)
Median lines of prior systemic therapy (range)*	4.5 (1–12)	4.0 (1–11)	4.0 (1–13)	3.0 (1–7)	3.0 (1–7)
Tumor type, n (%)					
Endometrium adenocarcinoma	2 (16.7)	0 (0)	3 (7.1)	7 (30.4)	0 (0)
NSCLC	1 (8.3)	5 (14.3)	2 (4.8)	6 (26.1)	5 (16.7)
Pancreatic cancer	1 (8.3)	17 (48.6)	4 (9.5)	6 (26.1)	6 (20.0)
RCC	1 (8.3)	1 (2.9)	1 (2.4)	0 (0)	0 (0)
Breast cancer	0 (0)	4 (11.4)	3 (7.1)	1 (4.3)	0 (0)
Melanoma	0 (0)	4 (11.4)	1 (2.4)	2 (8.7)	0 (0)
Bladder cancer	2 (16.7)	1 (2.9)	2 (4.8)	0 (0)	4 (13.3)
Gastric cancer	0 (0)	1 (2.9)	0 (0)	1 (4.3)	0 (0)
CRC	1 (8.3)	1 (2.9)	7 (16.7)	0 (0)	0 (0)
HNSCC	0 (0)	1 (2.9)	2 (4.8)	0 (0)	7 (23.3)
Other	4 (33.3)†	0 (0)	17 (40.5)‡	0 (0)	8 (26.7)§

*Lines of prior systemic therapy were not counted for one patient in part 1a group B. All regimen start dates are unknown for this patient.

†Cholangiocarcinoma, ovarian, and thyroid (n=1 each); other (n=1).

‡Adrenal, cervical, cholangiocarcinoma, mesothelioma, neuroendocrine, ovarian, and small cell lung cancer (n=1 each); salivary, thyroid, and uterine (n=2 each); other (n=4).

§Salivary gland (n=7); other (n=1).

CRC, colorectal cancer; ECOG PS, Eastern Cooperative Oncology Group performance status; HNSCC, head and neck squamous cell carcinoma; NSCLC, non-small cell lung cancer; RCC, renal cell carcinoma.

As of the data cut-off date (January 2, 2019), all patients in group A and 93 (97.9%) patients in group B had discontinued treatment. PD was the most common reason for treatment discontinuation: 83.3% in group A, 74.3% in groups A-1 and A-2, 76.2% in group B, 78.3% in groups B-1 and B-2, and 66.7% in groups B-3, B-4, and B-5.

### Safety

The MTD was not reached in group A and group B dose-escalation and dose-expansion groups, and therefore no MTD was defined for either the combination of itacitinib plus epacadostat or itacitinib plus parsaclisib. Doses reached and tested in the study were considered to be biologically effective for the study drugs, based on the pharmacodynamic effects of itacitinib and parsaclisib on immune-marker responses (see Plasma proteomic analysis section) and the effects of epacadostat on plasma kynurenine (see Plasma kynurenine analysis section).

#### Group A (itacitinib plus epacadostat)

No patient had a DLT in cycle 1. All patients had ≥1 TEAE (see online supplemental table 1). Treatment-related adverse events (TRAEs) were reported by seven (58.3%) patients in group A; the most common were pyrexia and vomiting (3 (25%) each), followed by chills and nausea (2 (16.7%) each) (table 2). TRAEs were reported by 31 (88.6%) patients in groups A-1 and A-2; the most common were fatigue (17, 48.6%), nausea (10, 28.6%), and vomiting (6, 17.1%). A total of 29 patients experienced ≥1 serious TEAE (7 (58.3%) in group A, 22 (62.9%) in groups A-1 and A2) (online supplemental table 2). Serious TEAEs considered by the investigator to be related to itacitinib and parsaclisib included aseptic meningitis (one in part 1a group B), one each of cardiomyopathy and pneumonitis in groups B-1 and B-2, and one each of lung infection and streptococcal bacteremia in group B-3, B-4, and B-5 population.

10.1136/jitc-2021-004223.supp1 Supplementary data

**Table 2:** Summary of TRAEs by MedDRA preferred term (≥10% of patients and the associated maximum severity of the safety evaluable population)

TRAE, n (%)	Group A: itacitinib+epacadostat				Group B: itacitinib+parsaclisib					
	Part 1a Dose-escalation Group A (n=12)		Part 1b Dose-expansion Groups A-1 and A-2 (n=35)		Part 1a Dose-escalation Group B (n=42)		Part 1b Dose-expansion Groups B-1 and B-2 (n=23)		Part 2 Dose-expansion Groups B-3, B-4, and B-5 (n=30)	
	Any grade	Grade ≥3	Any grade	Grade ≥3	Any grade	Grade ≥3*	Any grade	Grade ≥3	Any grade	Grade ≥3
Fatigue	1 (8.3)	0 (0)	17 (48.6)	2 (5.7)	9 (21.4)	0 (0)	8 (34.8)	0 (0)	9 (30.0)	2 (6.7)
Nausea	2 (16.7)	0 (0)	10 (28.6)	2 (5.7)	5 (11.9)	0 (0)	4 (17.4)	0 (0)	7 (23.3)	0 (0)
Vomiting	3 (25.0)	0 (0)	6 (17.1)	1 (2.9)	2 (4.8)	0 (0)	3 (13.0)	0 (0)	3 (10.0)	0 (0)
Decreased appetite	0 (0)	0 (0)	3 (8.6)	0 (0)	5 (11.9)	0 (0)	3 (13.0)	0 (0)	4 (13.3)	0 (0)
Anemia	0 (0)	0 (0)	2 (5.7)	0 (0)	0 (0)	0 (0)	1 (4.3)	0 (0)	5 (16.7)	1 (3.3)
Diarrhea	1 (8.3)	0 (0)	5 (14.3)	2 (5.7)	6 (14.3)	0 (0)	2 (8.7)	0 (0)	4 (13.3)	0 (0)
Pyrexia	3 (25.0)	0 (0)	5 (14.3)	1 (2.9)	9 (21.4)	0 (0)	3 (13.0)	0 (0)	1 (3.3)	0 (0)
Dizziness	0 (0)	0 (0)	1 (2.9)	0 (0)	0 (0)	0 (0)	0 (0)	0 (0)	3 (10.0)	0 (0)
Chills	2 (16.7)	0 (0)	1 (2.9)	0 (0)	5 (11.9)	0 (0)	2 (8.7)	0 (0)	0 (0)	0 (0)
Neck pain	0 (0)	0 (0)	0 (0)	0 (0)	0 (0)	0 (0)	0 (0)	0 (0)	3 (10.0)	0 (0)
Thrombocytopenia	0 (0)	0 (0)	4 (11.4)	1 (2.9)	0 (0)	0 (0)	0 (0)	0 (0)	0 (0)	0 (0)

*One patient experienced a DLT of grade 3 aseptic meningitis; the DLT resolved when the study drugs (itacitinib and parsaclisib) were withdrawn.

DLT, dose-limiting toxicity; MedDRA, Medical Dictionary for Regulatory Activities; TRAE, treatment-related adverse event.

A total of 10 patients discontinued treatment from all of group A (itacitinib plus epacadostat) due to TEAEs. One (8.3%) patient with anemia discontinued in dose-escalation group A. Nine (25.7%) patients discontinued treatment in dose-expansion groups A-1 and A-2 due to AST increase and fatigue (2 (5.7%) each), acute kidney injury, alanine aminotrasferase increase, atelectasis, blood ALP increase, blood bilirubin increase, blood creatinine increase, constipation, death, embolism, headache, respiratory failure, small intestinal obstruction, and tumor pain (1 (2.9%) each). Twelve (25.5%) patients in group A had a TEAE with a fatal outcome; all fatal events were considered unrelated to the study drugs by the treating investigators.

#### Group B (itacitinib plus parsaclisib or parsaclisib monotherapy)

One DLT was reported in a patient in dose-escalation group B (itacitinib 300 mg once a day plus parsaclisib 10 mg once a day dose level) of serious, grade 3 aseptic meningitis. The DLT resolved when the study drug was withdrawn and was considered related to itacitinib and parsaclisib.

Among all 95 patients in group B, 94 had ≥1 TEAE (see summary in online supplemental table 1). TRAEs occurred in 31 (73.8%) patients in part 1a group B, with fatigue and pyrexia (9 (21.4%) each) and diarrhea (6, 14.3%) being the most common. TRAEs occurred in 17 (73.9%) patients in groups B-1 and B-2, with the most common events being fatigue (8, 34.8%), nausea (4, 17.4%), and decreased appetite, pyrexia, and vomiting (3 (13.0%) each). TRAEs occurred in 24 (80.0%) patients in groups B-3, B-4, and B-5, with fatigue (9, 30.0%), nausea (7, 23.3%), and anemia (5, 16.7%) the most common. A total of 45 patients experienced ≥1 serious TEAE (20 (47.6%) in group B, 12 (52.2%) in groups B-1 and B-2, and 13 (43.3%) in groups B-3, B-4, and B-5). Serious TEAEs considered by the investigator to be related to itacitinib and parsaclisib included aseptic meningitis (one in part 1a group B), one each of cardiomyopathy and pneumonitis in groups B-1 and B-2, and one each of fatigue, pain, lung infection, streptococcal bacteremia, malignant neoplasm progression, dyspnea, and pleural effusion in the group B-3, B-4, and B-5 population.

Twenty-five patients experienced TEAEs that led to discontinuation of treatment in all of group B (itacitinib plus parsaclisib). Ten (23.8%) patients discontinued in dose-escalation group B; malignant neoplasm progression (2, 4.8%) was the most common reason for discontinuation. Six (26.1%) patients discontinued in dose-expansion groups B-1 and B-2, with nausea and vomiting (3 (13.0%) each) being the most common reason. Four (13.3%) patients discontinued itacitinib and five (16.7%) discontinued parsaclisib due to TEAEs in groups B-3, B-4, and B-5. Ascites, cerebrovascular accident, face swelling, facial pain, maculopapular rash, and small intestinal obstruction led to discontinuation of itacitinib and parsaclisib (groups B-3, B-4, and B-5), and pain led to discontinuation of parsaclisib only (group B-4). Fourteen (14.7%) patients in group B had a TEAE with fatal outcome; the treating investigators considered all fatal events unrelated to the study drugs.

In both groups A and B, no clinically meaningful trends were noted in hematology, clinical chemistry, coagulation, or urinalysis results. The mean changes from baseline in vital signs and ECG parameters were generally small. One patient in group A-2 and three patients in group B had elevations in both aminotransferases and bilirubin but did not meet Hy’s law criteria.

### Efficacy

#### Group A (itacitinib plus epacadostat)

**Figure 2:**
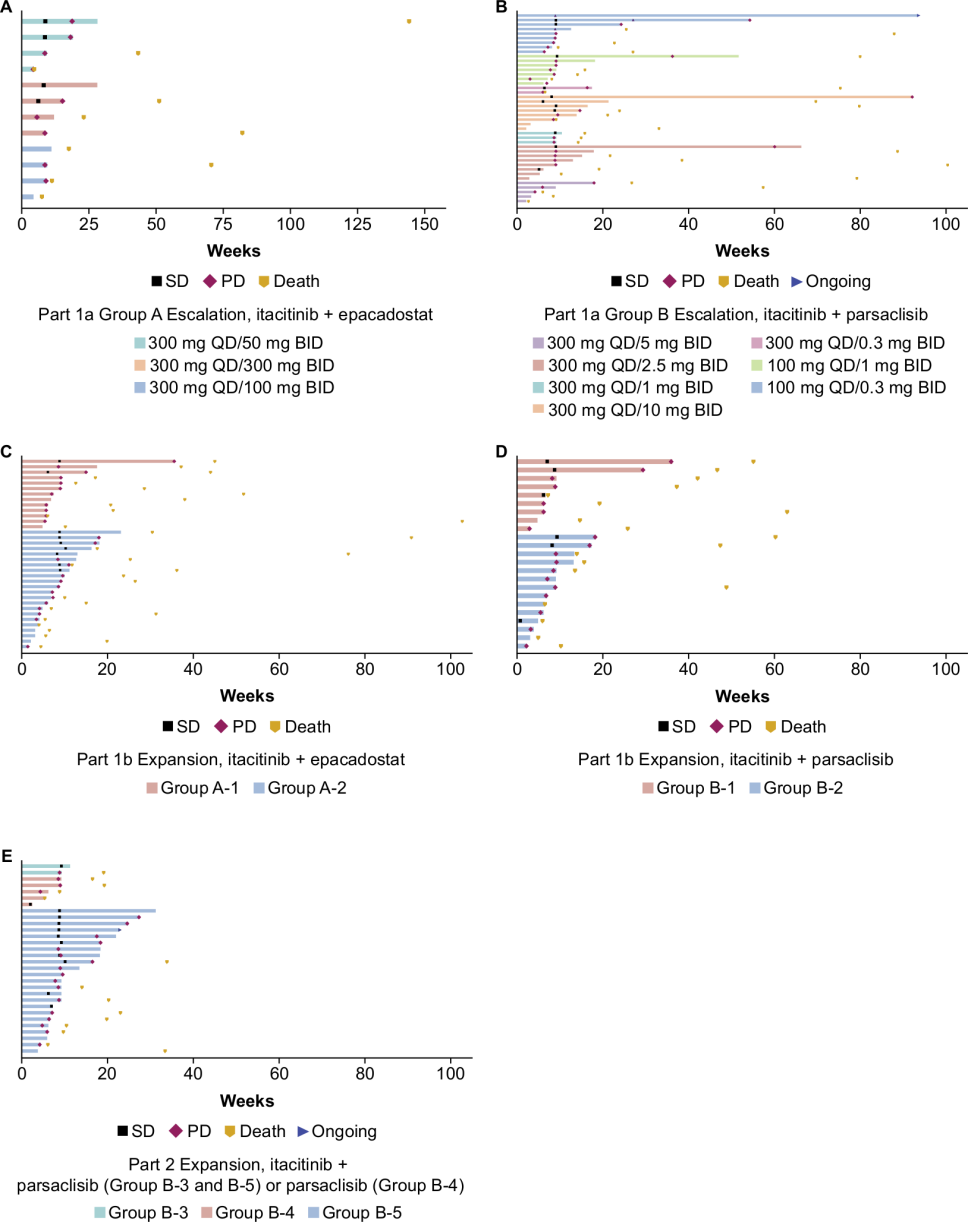
Swimmer plots of duration of treatment in the dose-escalation and dose-expansion groups (efficacy evaluable population). (A) Dose-escalation group A, (B) dose-expansion groups A-1 and A-2, (C) dose-escalation group B, (D) dose-expansion groups B-1 and B-2, and (E) dose-expansion groups B-3, B-4, and B-5. BID, two times per day; PD, progressive disease; QD, once a day; SD, stable disease.

No clinically relevant differences in overall response were observed between dose levels in dose-escalation group A and between dose-expansion groups A-1 and A-2 (data not shown). No patient had CR or PR in group A overall; four (33.3%) patients achieved stable disease in group A and nine (25.7%) patients in groups A-1 and A-2 (table 3). The median (95% CI) PFS was 2.0 months (1.3 to 4.2) in dose-escalation group A and 2.0 months (1.5 to 2.2) in dose-expansion groups A-1 and A-2. The duration of treatment is shown in figure 2A,B.

**Table 3:** Tumor response by RECIST (efficacy evaluable population)

Variable	Group A: itacitinib+epacadostat		Group B: itacitinib+parsaclisib		
	Part 1a Dose-escalation Group A (n=12)	Part 1b Dose-expansion Groups A-1 and A-2 (n=35)	Part 1a Dose-escalation Group B (n=42)	Part 1b Dose-expansion Groups B-1 and B-2 (n=23)	Part 2 Dose-expansion Groups B-3, B-4, and B-5 (n=30)
Best overall response, n (%)					
CR	0 (0)	0 (0)	0 (0)	0 (0)	0 (0)
PR	0 (0)	0 (0)	3 (7.1)*	0 (0)	0 (0)
SD	4 (33.3)	9 (25.7)	11 (26.2)	5 (21.7)	10 (33.3)
PD	6 (50.0)	20 (57.1)	22 (52.4)	14 (60.9)	16 (53.3)
NE	0 (0)	0 (0)	0 (0)	1 (4.3)	1 (3.3)
NA†	2 (16.7)	6 (17.1)	6 (14.3)	3 (13.0)	3 (10.0)
ORR‡, n (%)	0 (0)	0 (0)	3 (7.1)	0 (0)	0 (0)
95% CI for ORR§	0 to 26.46	0 to 10.00	1.50 to 19.48	0 to 14.82	0 to 11.57

*Tumor types of patients with PR were salivary gland cancer, HNSCC (nasopharynx), and other carcinoma.

† ‘NA’ includes any patients who did not have valid postbaseline overall response.

‡Patients who had best overall response of CR or PR.

§CI was calculated based on the exact method for binomial distributions.

CR, complete response; HNSCC, head and neck squamous cell carcinoma; NA, not assessed; NE, not estimable; ORR, objective response rate; PD, progressive disease; PR, partial response; RECIST, Response Evaluation Criteria in Solid Tumors; SD, stable disease.

#### Group B (itacitinib plus parsaclisib or parsaclisib monotherapy)

The ORR (95% CI) in dose-escalation group B was 7.1% (1.50 to 19.48); three patients (all at the itacitinib 100 mg once a day plus parsaclisib 0.3 mg once a day dose level) achieved PR (table 3). No patient in dose-expansion groups B-1 and B-2, or B-3, B-4, and B-5 had an objective response (table 3). Stable disease was achieved by 11 (26.2%) patients in group B, 5 (21.7%) patients in groups B-1 and B-2 (itacitinib 100 mg once a day plus parsaclisib 10 mg once a day), and 10 (33.3%) patients in groups B-3, B-4, and B-5 (itacitinib 100 mg once a day plus parsaclisib 0.3 mg once a day, or parsaclisib 0.3 mg once a day monotherapy). The median (95% CI) DOR for PR in dose-escalation group B was 6.3 (3.8, not estimable) months.

The median (95% CI) PFS was 2.1 (2.0 to 3.6) months in group B, 1.6 (1.4 to 2.0) months in groups B-1 and B-2, and 2.0 (1.8 to 3.7) months in groups B-3, B-4, and B-5. The duration of treatment is shown in figure 2C–E.

### Tumor lymphocyte response

#### Tumor IHC

**Figure 3:**
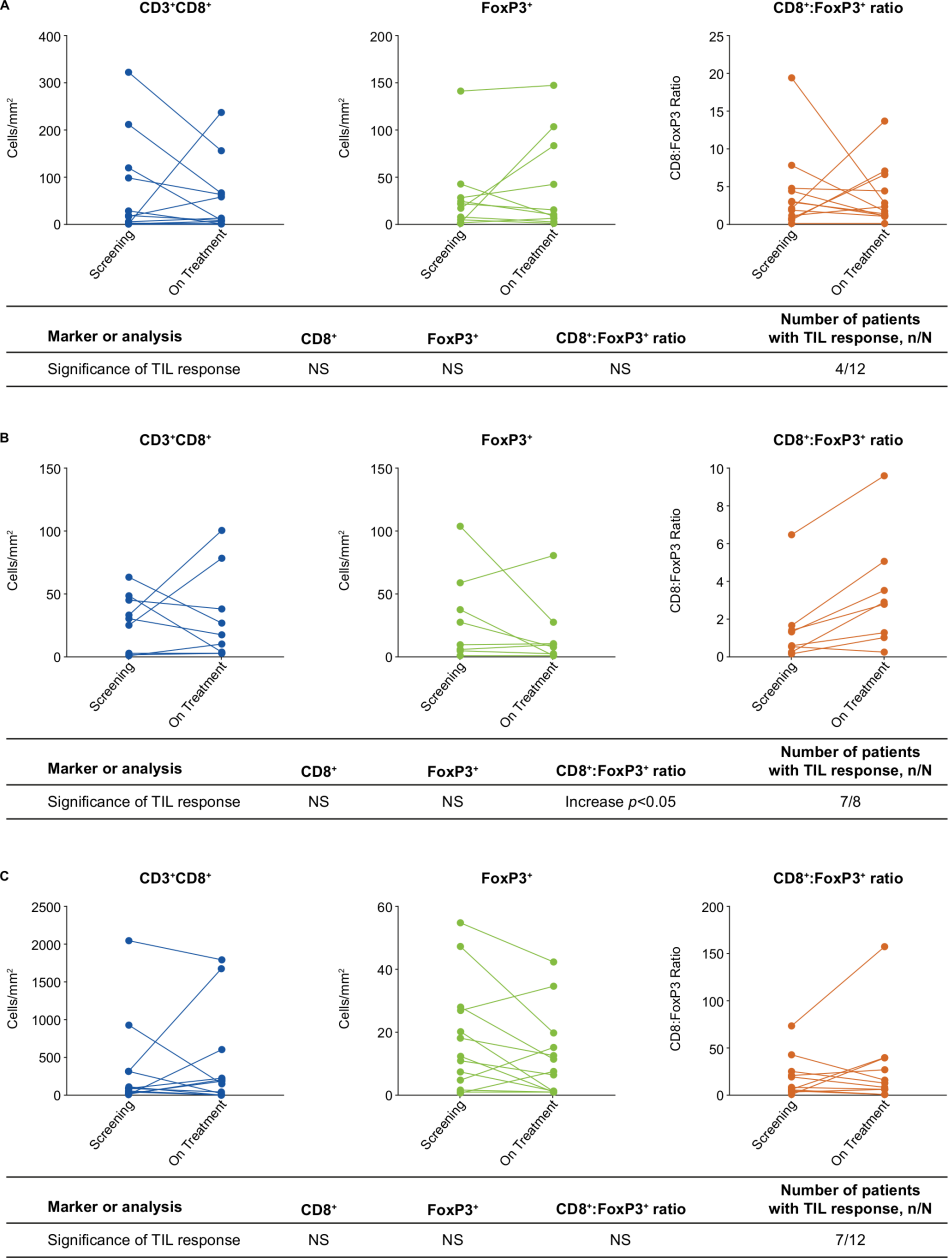
Immunohistochemistry analysis of T cell infiltration in (A) itacitinib plus epacadostat treatment samples (n=12), (B) itacitinib plus high-dose parsaclisib (1–10 mg) treatment samples (n=9; only 8 samples were available for TIL response assessment, since 1 sample failed FoxP3 analysis), and (C) itacitinib plus low-dose parsaclisib (0.3 mg) treatment samples (n=12). Comparisons were performed using Wilcoxon matched-pairs signed-rank test; changes were deemed significant at p<0.05. TIL responder was defined as ≥50% increase in the CD8^+^ to FoxP3^+^ cell ratio in the tumor compartment post-treatment versus baseline, as determined by immunohistochemistry. FoxP3, forkhead box protein 3; NS, not significant; TIL, tumor-infiltrating lymphocyte.

Changes in TIL and ratio of CD8^+^ effector to FoxP3^+^ Tregs (CD8^+^:FoxP3^+^) were determined by IHC using baseline and on-treatment tumor samples. In part 1 of the study, evaluable paired biopsy samples were available for 12 patients treated with itacitinib plus epacadostat and 9 patients treated with itacitinib plus high-dose (1–10 mg) parsaclisib. Treatment with itacitinib plus epacadostat demonstrated some increase in CD8^+^ T cell infiltration (figure 3A), with 4 of 12 patients classified as TIL responders. No significant change in the CD8:FoxP3 ratio was observed. There were no significant changes for tumor-infiltrating CD20 (B cell), CD45RO (activated/memory T cell), or CD68 (tissue macrophage) positive cell populations (data on file, Incyte). Treatment with itacitinib plus high-dose parsaclisib was associated with a decrease in stromal FoxP3^+^ cells, resulting in a significant increase in CD8:FoxP3 ratio (figure 3B). Although seven of eight patients were classified as TIL responders (ie, increase in CD8:FoxP3 ratio), an increased number of infiltrating CD8^+^ effector T cells was not observed. The treatment regimen also decreased the presence of CD68^+^ immunosuppressive TAMs in stromal (non-tumor), but not tumor, regions. There were no significant changes for tumor-infiltrating CD20 (B cell) or CD45RO (activated/memory T cell) positive cell populations (data on file, Incyte).

In part 2 of the study, evaluable paired biopsy samples were available for 12 patients treated with itacitinib plus low-dose (0.3 mg) parsaclisib. The IHC panel for part 2 included antibodies for phenotyping (CD3 (helper/effector T cells), CD8 (effector T cells), FoxP3 (Tregs)) and segmentation (panCK). The low-dose parsaclisib treatment regimen did not result in significant changes in intratumoral CD8^+^ cells, FoxP3^+^ cells, or CD8^+^:FoxP3^+^ ratio (figure 3C), whereas 7 of 12 patients were identified as TIL responders. Representative Multiplex IHC images in TCC and HNSCC are presented in online supplemental figure 3.

#### Plasma proteomic analysis

The presence of immune and non-immune plasma proteins was evaluated by proteomic analysis (1100 plasma analytes). Treatment with itacitinib plus epacadostat resulted in minor changes in six plasma proteins primarily involved in immune system regulation, suggesting that JAK1 inhibition may negatively affect the immune response (table 4, online supplemental figure 1). Proteins involved in the immune response that were downregulated with treatment included natural cytotoxicity triggering receptor 1 (NCR1 or CD335), interleukin 2 receptor alpha chain (IL2-RA or CD25), and tumor necrosis factor ligand superfamily (TNFSF) member 13B (B cell activating factor (BAFF)).

**Table 4:** Plasma proteins differentially expressed with itacitinib plus epacadostat or parsaclisib treatment

Plasma protein		Log_2_ fold change in protein level*
Treatment group A: itacitinib 300 mg plus epacadostat 300 mg (n=12)		
NCR1	Natural cytotoxicity triggering receptor 1 (CD335)	−0.52569
IL2-RA	Interleukin 2 receptor alpha chain (CD25)	−0.46984
TNFSF13B	TNFSF member 13B (BAFF or CD257)	−0.45461
CYR61	Cysteine-rich angiogenic inducer 61	0.464913
FGF-19	Fibroblast growth factor 19	1.133495
GAL	Galanin	1.134796
Treatment groups B-1 and B-2: itacitinib 300 mg plus parsaclisib 10 mg (n=12)		
CD160	Natural killer cell receptor, Ig superfamily member	−1.12002
CXCL13	CXC motif chemokine ligand 13	−0.93206
FCER2	Fc epsilon RII (CD23)	−0.84571
TNFRSF9	TNFRSF member 9 (CD137)	−0.79495
XCL1	C motif chemokine ligand 1 (lymphotactin)	−0.78599
NCR1	Natural cytotoxicity triggering receptor 1 (CD335)	−0.76039
FASLG	Fas ligand (TNFSF6 or CD95L)	−0.74582
SIGLEC6	Sialic acid binding Ig-like lectin 6	−0.64892
IL12	Interleukin 12	−0.6411
TRANCE†	TNF-related activation-induced cytokine	−0.63516
CD5	Lymphocyte antigen T1	−0.62866
FcRL2	Fc receptor-like protein 2	−0.6028
TNFB	TNF-beta (TNFSF1 or lymphotoxin-alpha)	−0.60078
IL-12B	Interleukin 12 subunit beta	−0.5857
FcRL6	Fc receptor-like protein 6	−0.5821
SIT1	Signaling threshold-regulating transmembrane adapter 1	−0.52955
LAIR-2	Leukocyte-associated immunoglobulin-like receptor 2 (CD306)	−0.50665
TNFRSF4	TNFRSF member 4 (OX40 receptor or CD134)	−0.4735
LILRB4	Leukocyte Ig-like receptor subfamily B member 4	−0.43298
TNFRSF13B	TNFRSF member 13B (CD267)	−0.42143
MUC-16	Mucin 16	0.408191
ST3GAL1	ST3 beta-galactoside alpha-2,3-sialyltransferase 1	0.425019
REG4	Regenerating islet-derived protein 4	0.428488
SMOC2	SPARC-related modular calcium-binding protein 2	0.437624
Flt3L	FMS-like tyrosine kinase 3 ligand	0.514995
GAL	Galanin	0.564124
THPO	Thrombopoietin	0.59239

*Baseline (C1D1) vs on-treatment (C2D1) samples were compared using a paired t-test, with changes considered significant at FDR p<0.05 and a log_2_ fold change >0.4 or <−0.4.

†Receptor activator of nuclear factor kappa-Β ligand (RANKL) or TNFSF11.

BAFF, B cell activating factor; C1D1, cycle 1 day 1; C2D1, cycle 2 day 1; CD, cluster of differentiation; FDR, false discovery rate; Ig, immunoglobulin; TNF, tumor necrosis factor; TNFRSF, tumor necrosis factor receptor superfamily; TNFSF, tumor necrosis factor ligand superfamily.

Itacitinib plus high-dose parsaclisib (10 mg) treatment demonstrated changes in 27 plasma proteins, of which 20 were downregulated on-treatment compared with baseline (table 4, online supplemental figure 1). Proteins decreased on-treatment were enriched for those involved in B cell, T cell, and natural killer (NK) cell proliferation and response. Proteins that were enriched on-treatment included (listed in descending order of increase) thrombopoietin, FMS-like tyrosine kinase 3 ligand (Flt3L), and SPARC-related modular calcium-binding protein 2 (SMOC2). The pharmacodynamic effects of low-dose parsaclisib (0.3 mg) were still evident, although reduced compared with high-dose parsaclisib (online supplemental figure 1).

#### Plasma kynurenine analysis

Plasma kynurenine concentration significantly decreased from baseline to cycle 2 in all patients treated with itacitinib (300 mg once a day) plus epacadostat (300 mg two times per day), with levels falling below the median healthy normal (1.5 µM) in 9 of 13 (69%) patients (online supplemental figure 2). Kynurenine levels generally rebounded from cycle 2 to 4, suggesting the pharmacodynamic response of IDO1 with epacadostat 300 mg two times per day was not durable.

## Discussion

This phase I platform study evaluated the safety, efficacy, and pharmacodynamics of itacitinib (JAK1 inhibitor) in combination with epacadostat (IDO1 inhibitor) or parsaclisib (PI3Kδ inhibitor) in patients with advanced solid tumors. The side effects of the treatment combinations used were manageable. One patient receiving itacitinib plus parsaclisib experienced a DLT of grade 3 aseptic meningitis, which resolved after both study drugs were withdrawn. Clinically meaningful efficacy was not observed with either itacitinib plus epacadostat, or itacitinib plus parsaclisib treatment combinations, regardless of the dose of parsaclisib evaluated; 13 (27.7%) and 26 (27.4%) patients had stable disease in groups A and B, respectively. No MTD was established for either of the treatment combinations, which could suggest the dosing was inadequate; however, based on preclinical data, adequate drug levels were achieved. Additionally, the doses of each drug have previously shown clinical activity. Itacitinib was clinically active in patients with myelofibrosis at doses of 200 mg two times per day or 600 mg once a day,^16^ and in acute graft versus host disease at doses of 200 or 300 mg once a day.^17^ Epacadostat doses were based on a phase I dose-escalation pharmacodynamic study^18 19^ and prior combination studies with pembrolizumab using epacadostat dose of 100 mg two times per day.^20 21^ Parsaclisib dosing in this study was based on emerging pharmacokinetic (PK) and pharmacodynamic data. In a phase I/II study, parsaclisib was clinically active in patients with B cell malignancies administered doses of 20 mg once a day for 9 weeks (followed by once weekly dosing).^22^

It should be noted that when epacadostat 100 mg two times per day was combined with pembrolizumab in patients with advanced melanoma, an interim analysis determined that an improved ORR was not expected and the study was terminated.^21^ Several other studies of epacadostat 100 mg two times per day in combination with pembrolizumab were subsequently reduced in scope and preliminary data did not show a consistent benefit.^20 23–25^ The patient groups assessed for plasma kynurenine response in our study received epacadostat 300 mg two times per day with itacitinib 300 mg once a day. A retrospective pooled analysis has shown that higher epacadostat doses (≥600 mg two times per day) may be necessary to overcome PD-1-associated kynurenine elevation during combination treatment.^26^ Thus, we cannot rule out the possibility that an antitumor immune response induced by itacitinib also increased IDO1 activity to levels not fully suppressed by epacadostat 300 mg two times per day. Prior PK analysis demonstrated that itacitinib could be administered with epacadostat or parsaclisib, as concomitant administration of itacitinib did not affect the PK profile of either epacadostat or parsaclisib, or vice versa (data on file, Incyte).

JAK1 inhibition combined with either IDO1 or PI3Kδ inhibition led to some changes in the TME and plasma proteins; however, overall this seemed to result in limited effects on antitumor immune responses in our study. Treatment with itacitinib plus epacadostat resulted in inconsistent changes to the CD8:FoxP3 ratio in the tumors (figure 3A). We also observed a decreased CD8:FoxP3 ratio in the stromal regions, and this effect was most evident in patients with a baseline CD8:FoxP3 ratio >1, where seven of seven patients with baseline stromal CD8:FoxP3 ratios >1 decreased on treatment (data on file, Incyte). However, since there was no consistent effect on TIL infiltration, proliferation, or STAT3 activity, the combination of JAK1 and IDO1 inhibition may be an unfavorable treatment combination for the development of an effective antitumor microenvironment.

Of note, CD68^+^ cells were decreased in the stromal regions of patients treated with itacitinib plus parsaclisib. CD68 is a marker of TAMs, and its presence in the tumor stroma has been suggested to be a prognostic factor for worse survival in some cancer types.^27–30^ Thus, this finding supports the notion that combined JAK1 plus PI3Kδ inhibition may support the development of some aspects associated with a favorable TME by decreasing the presence of immunosuppressive TAMs.

Itacitinib plus high-dose parsaclisib resulted in a significant decrease in stromal FoxP3^+^ and an increase in the CD8:FoxP3 ratio. In order to determine whether Treg function may be preferentially inhibited by low-dose parsaclisib, while sparing T-effector cell function, groups B3 and B5 were treated with parsaclisib 0.3 mg once a day. In this low-dose parsaclisib cohort, stromal FoxP3 cells were also decreased with a statistically non-significant trend toward an increase in the ratio of CD8:FoxP3. Collectively, since neither dose of parsaclisib induced an increase in tumorous CD8^+^ effector cells, the results suggest that the itacitinib plus parsaclisib combination is unable to enhance the antitumor immune response in the TME. Consistent with negative CD8^+^ effector T cell tumor infiltration biopsy analysis, no patient in the dose-expansion group had an objective response.

The effects of study treatments were further evaluated by plasma protein analysis. The combination of itacitinib (300 mg) plus epacadostat (300 mg) resulted in changes in a small number of plasma proteins (six proteins overall). Three proteins with decreased expression were NCR1, IL2-RA, and TNFSF13B (BAFF); their downregulation is anticipated to impair NK cell, T cell, and B cell function, respectively. In contrast to the modest effects of itacitinib plus epacadostat, treatment with itacitinib (300 mg) plus parsaclisib (10 mg) resulted in changes to a larger set of proteins (27 proteins overall). The majority of proteins that were modulated with the combination of itacitinib plus parsaclisib are involved in the regulation of T cell, NK cell, and B cell activity, indicating that this treatment combination likely suppresses at least some aspects of immune activation. Proteins with decreased expression involved in T cell growth and function include CD160, TNFRSF9, XCL1, FASLG, interleukin 12 (IL-12), and IL-12 subunit B, Fc receptor-like protein 6 (FcRL6), and TNFRSF4 (OX40, a T cell secondary costimulatory immune checkpoint receptor). This broad array of proteins downregulated following treatment with itacitinib plus parsaclisib is anticipated to impair T cell activity and may correlate with the negative T cell tumor infiltration biopsy analysis in our study. Proteins involved in B cell function with decreased expression following this combination included CXCL13 (B cell chemokine), Fc receptor-like protein 2 (FcRL2), and TNFRSF13B; this result is consistent with demonstrated clinical activity of parsaclisib in B cell malignancies.^22^

Taken together, these pharmacodynamic data demonstrate that the predictions based on preclinical studies^5 15^ were not borne out in the clinic. In our clinical study, JAK1 inhibition combined with either IDO1 or PI3Kδ inhibition did not lead to enhanced immune activation as evidenced by a lack of CD8^+^ T-effector cell infiltration into the tumor microenvironment. These results are further supported by the lack of clinical efficacy observed in either treatment group. Plasma proteomic analysis demonstrated that the combination of itacitinib plus parsaclisib downregulated 20 proteins, which are mostly involved in immune cell (ie, NK, B, and T cell) activity and function. Suppression of T lymphocytes with itacitinib plus parsaclisib may correlate with the negative T cell tumor infiltration observed in our study. Further investigations are needed to fully evaluate combinations of targeted agents that may elicit antitumor immune responses.
