# Correction: Exploring the safety, effect on the tumor microenvironment, and efficacy of itacitinib in combination with epacadostat or parsaclisib in advanced solid tumors: a phase I study

**DOI:** 10.1136/jitc-2021-004223corr1

**Published:** 2022-10-25

Naing A, Powderly JD, Nemunaitis JJ, *et al*. Exploring the safety, effect on the tumor microenvironment, and efficacy of itacitinib in combination with epacadostat or parsaclisib in advanced solid tumors: a phase I study. *J Immunother Cancer* 2022;10:e004223. doi: 10.1136/jitc-2021-004223

The last sentence of the section titled 'Group A (itacitinib plus epacadostat)' has been updated to 'Serious TEAEs considered by the investigator to be related to itacitinib and parsaclisib included aseptic meningitis (one in part 1a group B), one each of cardiomyopathy and pneumonitis in groups B-1 and B-2, and one each of lung infection and streptococcal bacteremia in group B-3, B-4, and B-5 population'.

