# Supplementary material for: Exploring the safety, effect on the tumor microenvironment, and efficacy of itacitinib in combination with epacadostat or parsaclisib in advanced solid tumors: a phase I study
Source: J Immunother Cancer. 2022 Mar 14;10(3):e004223. doi: 10.1136/jitc-2021-004223 (PMC8921936; doi:10.1136/jitc-2021-004223)

SUPPLEMENTAL MATERIAL

Phase I study exploring the safety, effect on the tumor microenvironment, and efficacy of itacitinib in combination with epacadostat or parsaclisib in advanced solid tumors

Aung Naing,[1*] John D Powderly,[2] John J Nemunaitis,[3†] Jason J Luke,[4] Aaron S Mansfield,[5] Wells A Messersmith,[6] Solmaz Sahebjam,[7] Patricia M LoRusso,[8] Ignacio Garrido-Laguna,[9] Lance Leopold,[10] Ryan Geschwindt,[10] Kai Ding,[11] Michael Smith,[10] Jordan D Berlin[12]

[1]Department of Investigational Cancer Therapeutics, MD Anderson Cancer Center, Houston, Texas, USA; [2]Cancer Research Clinic, Carolina Bio-oncology Institute, Huntersville, North Carolina, USA; [3]University of Toledo College of Medicine, Toledo, Ohio, USA; [4]Division of Hematology/Oncology, University of Pittsburgh Medical Center, Pittsburgh, Pennsylvania, USA; [5]Oncology, Mayo Clinic, Rochester, Minnesota, USA; [6] School of Medicine, University of Colorado, Aurora, Colorado, USA; [7]Clinical Research Unit, Moffitt Cancer Center, Tampa, Florida, USA; [8]Yale School of Medicine, Yale Cancer Center, New Haven, Connecticut, USA; [9]University of Utah School of Medicine, Huntsman Cancer Institute, Salt Lake City, Utah, USA; [10]Immuno-Oncology, Incyte Corporation, Wilmington, Delaware, USA; [11]Biostatistics, Incyte Corporation, Wilmington, Delaware, USA; [12]Division of Hematology/Oncology, Vanderbilt University, Nashville, Tennessee, USA

Current affiliation: [†]Gradalis, Inc., Carrollton, Texas, USA

***Correspondence:** Dr Aung Naing, MD Anderson Cancer Center, Unit Number 455, 1515 Holcombe Blvd, Houston, Texas 77030, USA. Tel: +1 713-563-0181.

Email: anaing@mdanderson.org.

Supplemental table 1 Summary of TEAEs by MedDRA preferred term (>15% of patients and the associated maximum severity of the safety evaluable population)

	Group A: itacitinib + epacadostat				Group B: itacitinib + parsaclisib					
	Part 1a Dose-escalation Group A (N=12)		Part 1b Dose-expansion Groups A-1 and A-2 (N=35)		Part 1a Dose-escalation Group B (N=42)		Part 1b Dose-expansion groups B-1 and B-2 (N=23)		Part 2 Dose-expansion Groups B-3, B-4, and B-5 (N=30)	
**TEAE, n (%)**	**Any grade**	**Grade ≥3**	**Any grade**	**Grade ≥3**	**Any grade**	**Grade ≥3**	**Any grade**	**Grade ≥3**	**Any grade**	**Grade ≥3**
Fatigue	4 (33.3)	0 (0)	20 (57.1)	2 (5.7)	16 (38.1)	2 (4.8)	12 (52.2)	1 (4.3)	10 (33.3)	2 (6.7)
Nausea	3 (25.0)	0 (0)	17 (48.6)	2 (5.7)	11 (26.2)	0 (0)	9 (39.1)	3 (13.0)	12 (40.0)	0 (0)
Vomiting	4 (33.3)	0 (0)	13 (37.1)	2 (5.7)	9 (21.4)	2 (4.8)	9 (39.1)	3 (13.0)	7 (23.3)	0 (0)
Decreased appetite	1 (8.3)	0 (0)	9 (25.7)	0 (0)	11 (26.2)	0 (0)	11 (47.8)	1 (4.3)	5 (16.7)	0 (0)
Abdominal pain	2 (16.7)	1 (8.3)	8 (22.9)	3 (8.6)	7 (16.7)	1 (2.4)	4 (17.4)	2 (8.7)	5 (16.7)	0 (0)
Anemia	1 (8.3)	1 (8.3)	8 (22.9)	2 (5.7)	8 (19.0)	3 (7.1)	3 (13.0)	3 (13.0)	7 (23.3)	2 (6.7)
Back pain	1 (8.3)	0 (0)	8 (22.9)	0 (0)	5 (11.9)	1 (2.4)	5 (21.7)	0 (0)	3 (10.0)	0 (0)
Constipation	4 (33.3)	0 (0)	8 (22.9)	0 (0)	9 (21.4)	0 (0)	6 (26.1)	0 (0)	7 (23.3)	0 (0)
Dehydration	1 (8.3)	0 (0)	7 (20.0)	1 (2.9)	7 (16.7)	2 (4.8)	7 (30.4)	2 (8.7)	4 (13.3)	3 (10.0)
Diarrhea	2 (16.7)	0 (0)	7 (20.0)	3 (8.6)	14 (33.3)	1 (2.4)	3 (13.0)	0 (0)	6 (20.0)	0 (0)
Pyrexia	4 (33.3)	0 (0)	7 (20.0)	1 (2.9)	12 (28.6)	0 (0)	5 (21.7)	0 (0)	3 (10.0)	0 (0)

Naing A, *et al. J Immunother Cancer* 2022; 10:e004223. doi: 10.1136/jitc-2021-004223

Malignant neoplasm progression	2 (16.7)	2 (16.7)	5 (14.3)	5 (14.3)	5 (11.9)	4 (9.5)	4 (17.4)	4 (17.4)	2 (6.7)	2 (6.7)
Cough	3 (25.0)	0 (0)	4 (11.4)	0 (0)	8 (19.0)	0 (0)	4 (17.4)	0 (0)	6 (20.0)	0 (0)
Dizziness	2 (16.7)	0 (0)	4 (11.4)	0 (0)	1 (2.4)	0 (0)	1 (4.3)	0 (0)	5 (16.7)	0 (0)
Pneumonia*	2 (16.7)	2 (16.7)	3 (8.6)	1 (2.9)	1 (2.4)	1 (2.4)	1 (4.3)	NR	1 (3.3)	1 (3.3)
Anxiety	2 (16.7)	0 (0)	2 (5.7)	0 (0)	1 (2.4)	0 (0)	NR	NR	1 (3.3)	0 (0)
Chills	2 (16.7)	0 (0)	2 (5.7)	0 (0)	5 (11.9)	0 (0)	3 (13.0)	0 (0)	2 (6.7)	0 (0)
Insomnia	3 (25.0)	0 (0)	2 (5.7)	0 (0)	2 (4.8)	0 (0)	1 (4.3)	0 (0)	1 (3.3)	0 (0)
Ear pain	2 (16.7)	0 (0)	NR	NR	NR	NR	NR	NR	NR	NR
Muscle spasms	2 (16.7)	0 (0)	NR	NR	1 (2.4)	0 (0)	NR	NR	NR	NR
Myalgia	NR	NR	5 (14.3)	0 (0)	4 (9.5)	0 (0)	5 (21.7)	0 (0)	3 (10.0)	0 (0)
Blood alkaline phosphatase increased	1 (8.3)	0 (0)	2 (5.7)	0 (0)	5 (11.9)	2 (4.8)	4 (17.4)	1 (4.3)	3 (10.0)	0 (0)
Dyspnea	1 (8.3)	0 (0)	5 (14.3)	2 (5.7)	7 (16.7)	2 (4.8)	4 (17.4)	1 (4.3)	3 (10.0)	2 (6.7)
Headache	1 (8.3)	0 (0)	4 (11.4)	1 (2.9)	4 (9.5)	0 (0)	NR	NR	5 (16.7)	0 (0)

*Severity grade was missing for the patient who experienced a TEAE of pneumonia in Part 1b dose-expansion Group B-1 and B-2.

MedDRA, Medical Dictionary for Regulatory Activities; TEAE, treatment-emergent adverse event; NR, not reported.

Supplemental table 2 Serious TEAE by MedDRA preferred term (≥5% of patients of any treatment group of the safety evaluable population)

	Group A: itacitinib + epacadostat		Group B: itacitinib + parsaclisib		
**Serious TEAE by MedDRA preferred term*, n (%)**	**Part 1a Dose-escalation Group A (N=12)**	**Part 1b Expansion Groups A-1 and A-2 (N=35)**	**Part 1a Dose-escalation Group B (N=42)**	**Part 1b Expansion Groups B-1 and B-2 (N=23)**	**Part 2 Expansion Groups B-3, B-4, and B-5 (N=30)**
Anemia	1 (8.3)	1 (2.9)	0 (0)	2 (8.7)	0 (0)
Anxiety	1 (8.3)	0 (0)	0 (0)	0 (0)	0 (0)
Biliary tract infection	1 (8.3)	0 (0)	0 (0)	0 (0)	0 (0)
Deep vein thrombosis	1 (8.3)	0 (0)	0 (0)	0 (0)	0 (0)
Encephalopathy	1 (8.3)	0 (0)	0 (0)	0 (0)	0 (0)
Pneumonia	1 (8.3)	1 (2.9)	1 (2.4)	0 (0)	0 (0)
Procedural hemorrhage	1 (8.3)	0 (0)	0 (0)	0 (0)	0 (0)
Pulmonary embolism	1 (8.3)	0 (0)	0 (0)	2 (8.7)	0 (0)
Respiratory failure	1 (8.3)	1 (2.9)	0 (0)	0 (0)	0 (0)
Abdominal pain	0 (0)	3 (8.6)	1 (2.4)	2 (8.7)	0 (0)
Small intestinal obstruction	0 (0)	3 (8.6)	0 (0)	0 (0)	1 (3.3)
Diarrhea	0 (0)	2 (5.7)	0 (0)	0 (0)	0 (0)
Dyspnea	0 (0)	2 (5.7)	0 (0)	1 (4.3)	2 (6.7)
Nausea	0 (0)	2 (5.7)	0 (0)	3 (13.0)	1 (3.3)

Naing A, *et al. J Immunother Cancer* 2022; 10:e004223. doi: 10.1136/jitc-2021-004223

Vomiting	0 (0)	2 (5.7)	1 (2.4)	3 (13.0)	1 (3.3)
Dehydration	0 (0)	1 (2.9)	0 (0)	3 (13.0)	0 (0)
Tumor pain	0 (0)	1 (2.9)	0 (0)	2 (8.7)	0 (0)
Acute kidney injury	0 (0)	1 (2.9)	3 (7.1)	1 (4.3)	1 (3.3)
Lung infection	0 (0)	0 (0)	0 (0)	1 (4.3)	2 (6.7)

*Preferred terms are arranged by decreasing order of frequency of TEAEs in Part 1a, Group A. TEAE was defined as any adverse event either reported for the first time or worsening of a pre-existing event after first dose of study drug and until 30 days after the last dose of study drug.

MedDRA, Medical Dictionary for Regulatory Activities; TEAE, treatment-emergent adverse event.

Supplemental figure 1 Heat map of average Log2 fold change of all proteins identified by paired t-test to be differentially expressed (FDR ≤0.05 and Log2 fold change >0.4 or <−0.4) between C1D1 and C2D1 in any treatment group.

Delta 0.3 mg QD: itacitinib 100 mg (n=23 [Group B-3 and B-5]) or 300 mg (n=2 [Part 1a Group B]) plus parsaclisib 0.3 mg; Delta 10 mg QD: itacitinib 300 mg plus parsaclisib 10 mg (n=12 [Group B-1 and B-2]); Epacadostat: itacitinib 300 mg plus epacadostat 300 mg (n=12 [Group A-1 and A-2]).

C1D1, cycle 1 day 1; C2D1, cycle 2 day 1; delta, parsaclisib; FDR, false discovery rate; QD, once daily.

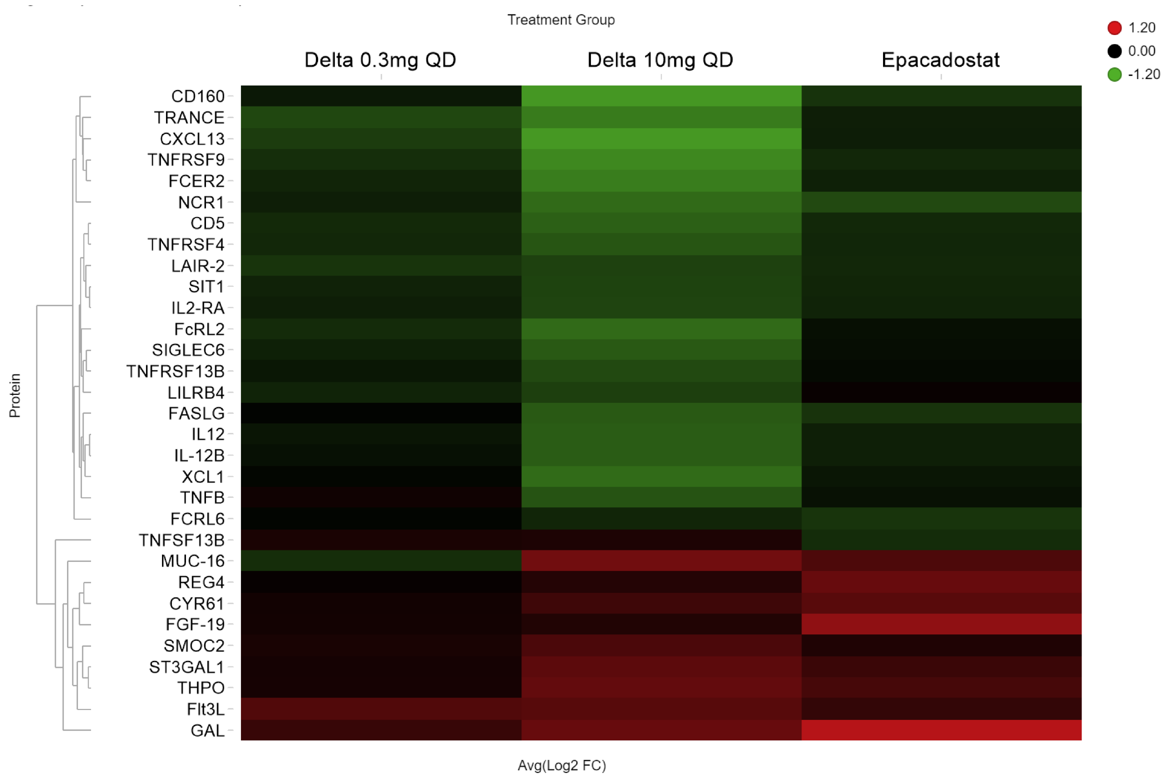

6

Supplemental figure 2 Kynurenine levels were analyzed in baseline (C1D1) versus on-treatment (itacitinib 300 mg QD + epacadostat 300 mg BID) plasma samples (C2D1, C4D1) from Groups A-1 (n=5) and A-2 (n=8).

BID, twice daily; C1D1, cycle 1 day 1; C2D1, cycle 2 day 1; C4D1, cycle 4 day 1; PD, progressive disease; QD, once daily; SD, stable disease.

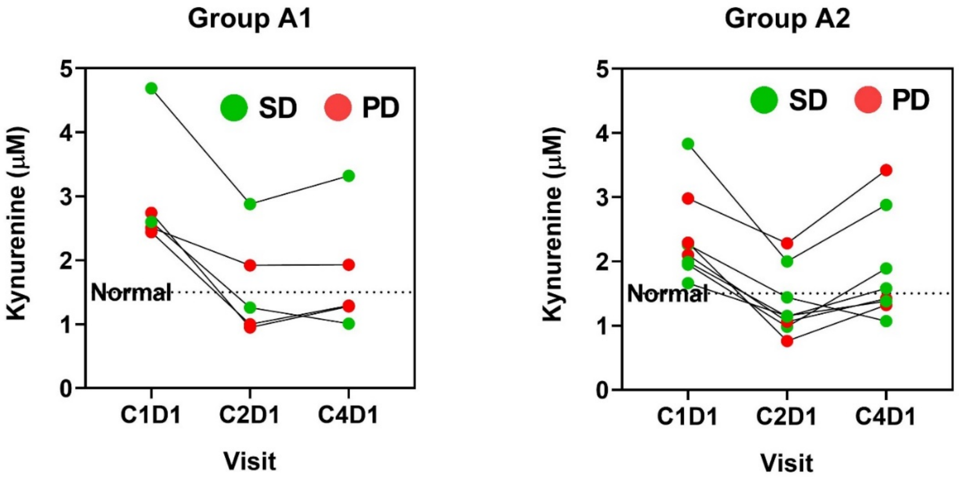

Supplemental figure 3 Representative immunohistochemistry staining for CD8^{+} T cells at screening compared with on-treatment with itacitinib plus low-dose parsaclisib (0.3 mg). Tumor infiltrating CD8^{+} and FoxP3^{+} (forkhead box protein 3) positive cells were quantified by counting cells present within the tumor as assessed by co-localization with pan-cytokeratin staining. (A) Patient with transitional cell carcinoma of urinary tract: 318 CD8^{+}/mm^{2} cells at screening (left panel) versus 1681 CD8^{+}/mm^{2} cells on-treatment (right panel). (B) Patient with head and neck squamous cell carcinoma: 932 CD8^{+}/mm^{2} at screening (left panel) versus 152 CD8^{+}/mm^{2} cells on-treatment (right panel).

Orange = panCK (segmentation); green = CD8 (effector T cells); magenta = FoxP3 (regulatory T cells).

A

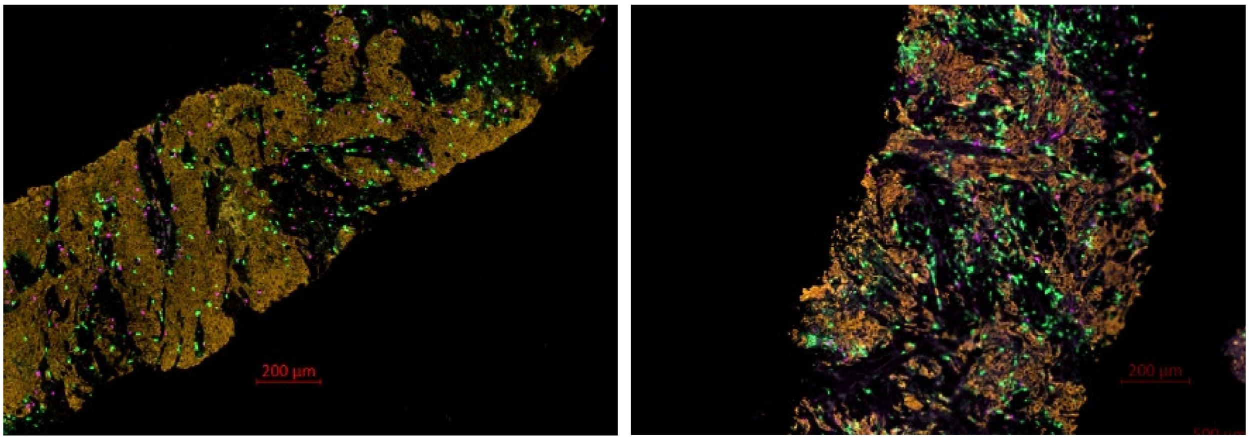

B

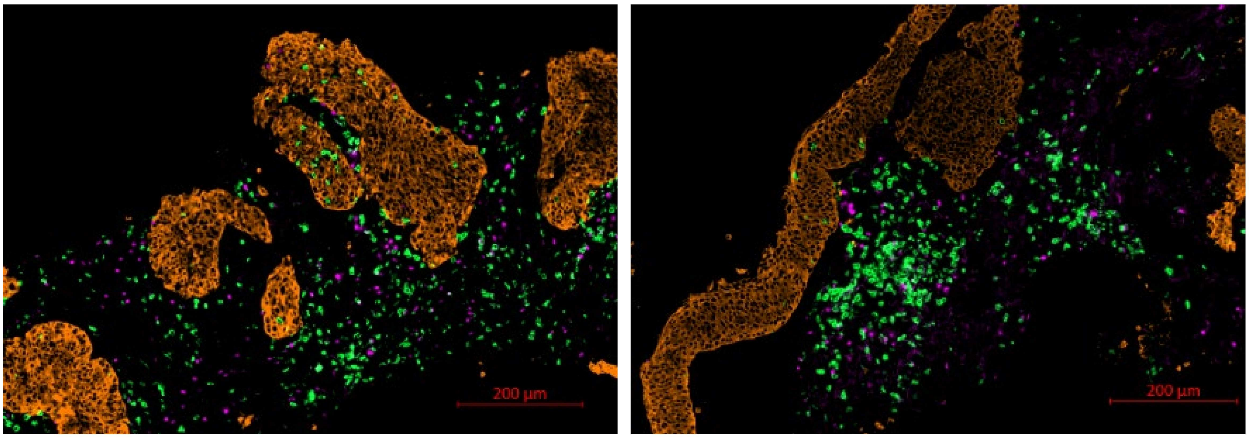

Supplement: Supplementary data [file jitc-2021-004223supp001.pdf]
